# Exploring the nutritional and sensory potential of karonda fruit: Physicochemical properties, jam production, and quality evaluation

**DOI:** 10.1002/fsn3.3619

**Published:** 2023-09-08

**Authors:** Nagina Rafique, Turfa Mamoona, Nazish Ashraf, Sheraz Hussain, Faisal Ahmed, Tawaf Ali Shah, Ahmad Mohammad Salamatullah, Amare Bitew Mekonnen, Mohammed Bourhia

**Affiliations:** ^1^ Department of Food Science and Technology University of Poonch Rawalakot Azad Jammu and Kashmir Pakistan; ^2^ Department of Agriculture Engineering and Food Science Shandong University of Technology Zibo China; ^3^ Department of Food Science & Nutrition, College of Food and Agricultural Sciences King Saud University Riyadh Saudi Arabia; ^4^ Department of Biology Bahir Dar University Bahir Dar Ethiopia; ^5^ Department of Chemistry and Biochemistry, Faculty of Medicine and Pharmacy Ibn Zohr University Laayoune Morocco

**Keywords:** anthocyanin, antioxidant properties, apple, karonda, nutritional value

## Abstract

Karonda is an indigenous berry fruit known for its unique sour taste and high nutritional value. The lack of awareness portrays the fruit as undervalued and neglected, despite its vast nutritional benefits. The study aimed to explore the physicochemical properties of fresh and dried karonda fruit and its application in formulating a jam product. The physicochemical parameters, including pH, acidity, reducing sugars, moisture content, ash content, and others, were analyzed for both fresh and dried fruits. The phytochemical characteristics, such as vitamin C, antioxidant activity, total phenolic content, flavonoids, and anthocyanin content, were examined. The fruit extract was also subjected to antibacterial test using the well plate method. The fresh karonda berries have the highest levels of vitamin C, total phenolic, and anthocyanin contents, which can enhance the immune system and improve overall health. Jam formulations were created using varying proportions of karonda and apple pulp. These formulations were subsequently analyzed for their physicochemical, phytochemical, and sensory quality attributes. The results indicated that the pH, moisture content, ash content, ascorbic acid content, total phenolics, total flavonoid content, and antioxidant activity of the jams fell within the acceptable range as outlined by the Codex Alimentarius. Furthermore, the inclusion of apple pulp can enhance the taste and color of the jam while preserving its nutritional value. The sensory evaluation results revealed that T3, consisting of 50% karonda and 50% apple, followed by T4, comprising 25% karonda and 75% apple, were favored in terms of taste and color. This research offers significant insights for both the food industry and consumers, emphasizing the karonda fruit's potential as a valuable source of phytochemical compounds and its possible utilization in the creation of jams and other food products. This discovery promotes the consumption of this indigenous fruit due to its nutritional value and potential health benefits.

## Graphical abstract

Karonda is an indigenous berry fruit, known for its unique sour taste and high nutritional value. The study aimed to explore the physiochemical properties of fresh and dried karonda fruit and its use in jam production. This research provides valuable information about the nutritional value of fresh and dried karonda fruit, which can be used to promote the consumption of this indigenous fruit.

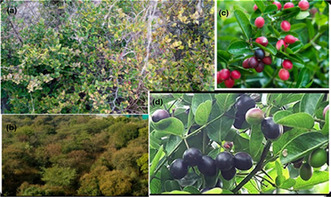

## 1 INTRODUCTION

**FIGURE 1:**
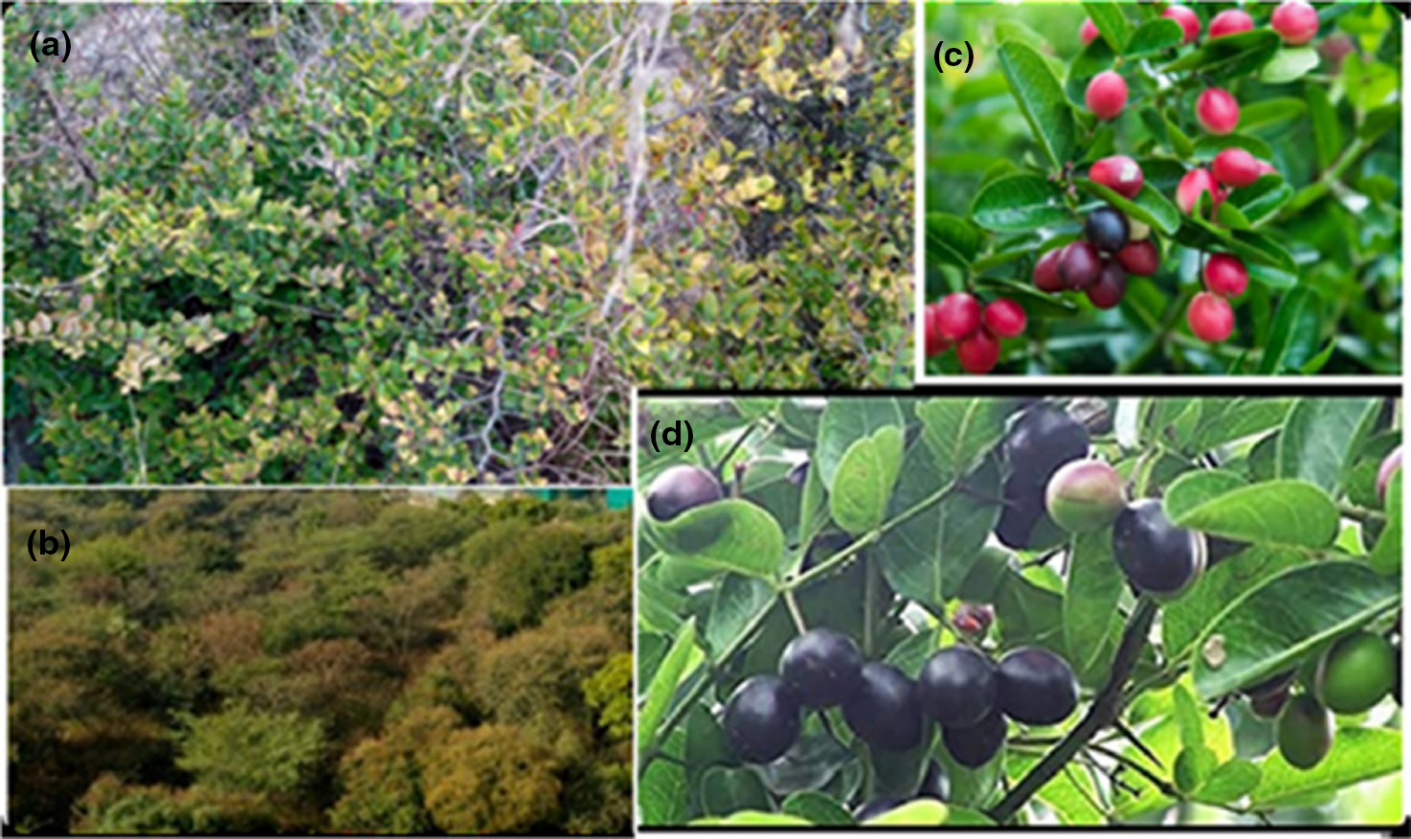
*Carissa spinarum* a typical plant (a, b) and fruit‐bearing branches showing partially ripened to fully ripened fruits (c, d).

Many wild edible plants are nutrient‐dense and can be utilized to enhance the nutritional needs of both humans and cattle, particularly in terms of vitamins and minerals. Underutilized plant species possess considerable potential to contribute to food security, health (both nutritional and medicinal), revenue generation, and environmental benefits. However, they are not being fully utilized to their maximum potential (Arora, 2014). Karonda is a robust, evergreen, prickly shrub that is commonly cultivated in Asia. It belongs to the Apocynaceae family, which also encompasses the Oleander and the Vinca. The plant is indigenous to Pakistan and can also be found in Sri Lanka, Indonesia, Malaysia, Myanmar, India, the Siwalik Hills, the Western Ghats, Nepal, Afghanistan, Java, Australia, and South Africa, where it is referred to as Christ thorn and karonda. In Azad Kashmir, Pakistan, it is known as karonda (Khan et al., 2008). Carissa is a genus comprising approximately 25 species, 5 of which have their origins in Azad Kashmir, Pakistan. These species include *Carissa carandas* L., *Carissa congesta* L., *Carissa spinarum* L., *Carissa edulis*, and *Carissa grandiflora*. The plant is a small shrub or bush that can grow up to three meters tall. It produces small white flowers and red or black berries (Figure 1). The fruit is typically round or oval in shape, about the size of a cherry, ripens in the summer, and has a sour taste (Meena et al., 2020). *Carissa spinarum* was widely distributed throughout the collection site and has tremendous nutritional and medicinal potentials. The pharmacological studies by in vitro and in vivo experiments on *C. spinarum* discovered its anticonvulsant, anthelmintic, anti‐arthritic, antimicrobial, antidiabetic, anti‐inflammatory, hepatoprotective, antioxidant, vasorelaxant, antitumor, antihypertensive, wound‐healing, antipyretic effects, and anti‐venom properties (Sharma et al., 2023).

The karonda fruit is relatively unknown outside of Pakistan and India; however, it is gaining popularity due to its potential health benefits. The plant is commonly cultivated in home gardens and commercially grown in certain regions. Karonda fruit is renowned for its high nutritional value, being a rich source of vitamin C, vitamin A, and other antioxidants such as phenol, DPPH scavenging activity, flavonoids, tannins, and anthocyanin. Additionally, it is believed to possess anti‐inflammatory and antimicrobial properties. Traditionally, the fruit has been utilized in ayurvedic medicine to address various ailments, including digestive issues and respiratory problems (Meghwal et al., 2014). The plants such as karonda, blackberry, and mulberry contained the higher proportion of natural active anthocyanin content, and the extracted pigment has been reported for its use in the development of different food products (Singh et al., 2020).

Carissa species has been extensively employed for the treatment of numerous ailments. Flatulence, indigestion, acidity, and sores are all indicative of a compromised immune system. Various human ailments include diarrhea, stomachic issues, anorexia, intermittent fever, mouth ulcers, sore throat, syphilitic discomfort, burning sensations, scabies, and epilepsy (Verma et al., 2015). The *Carissa* fruits are rich in vitamin C, dietary fiber, carbohydrates, lipids, proteins, and micro‐elements. Most of the *Carissa* species are used to treat various diseases traditionally, such as headaches, chest pain, rheumatism, gonorrhea, stomach pain, syphilis, hepatitis, edema, rabies, asthma, and cardiac diseases (Dhatwalia et al., 2021).

A study published in the Journal of Ethnopharmacology in 2013 discovered that the fruit had a significant impact on lowering blood sugar levels in diabetic rats (Pandey et al., 2013). Another study, published in the same journal in 2014, revealed that karonda extract exhibited a positive impact on liver function in rats (Pandey et al., 2014).

Karonda is a delicious appetizer. Fruit is commonly preserved through pickling before it ripens. Owing to the substantial pectin content found in ripe karonda fruit, it is also utilized in the creation of jelly, jam, squash, sauces, pies, syrup, tarts, and chutney, all of which are highly sought after on the global market (Wani et al., 2013). When the ripe fruit is cooked, it exudes a gummy latex; however, upon cooling, it releases a vibrant red juice that turns clear, rendering it an exquisite and delicious summer beverage. The juice derived from ripe karonda is easily digestible, remarkably refreshing, delightful, and nutritionally superior to numerous synthetic and carbonated beverages (Navya et al., 2020).

Kashmir is bestowed with abundant plant biodiversity, and its tribal regions are rich with the wealth of underutilized natural vegetation. These crops are cultivated and locally consumed. They offer numerous advantages, as they are easy to grow and can produce a crop even under adverse soil and climatic conditions. Consequently, the exploitation of underutilized plants could provide a solution to the social problem of food insecurity. Karonda is an arid resin fruit that is highly produced in Kashmir. It is rich in vitamins and minerals and serves as the primary source of iron. Karonda can be grown in various types of soil and is cost‐effective to produce. However, the local people who live in the study areas consume the fruit without being aware of its nutrient content. It is also utilized as traditional medicine in Azad Jammu and Kashmir (Pakistan) for treating various ailments such as fever, cold, cough, and jaundice. In recent years, research has been conducted on the potential health benefits of karonda. Therefore, this research was planned to investigate the physicochemical qualities and nutritional values of karonda fruits from different geographical locations in Hajira, Azad Kashmir. Hence, the objective was to evaluate the nutritional potential of karonda fruit products.

## 2 MATERIALS AND METHODS

### 2.1 Sample collection

The study was conducted during March–August 2022, in the Laboratory of Food Science and Technology at the University of Poonch, Rawalakot, AJK. The fresh samples of karonda fruit were collected randomly from the side area of Hajira, Azad Jammu and Kashmir (at the latitude 33° 46′ 18.12″ N, longitude 73° 73° 53′ 45.96″ E and altitude of 3168 feet) and were transferred to the laboratory of Food Science and Technology. The samples of mature fruit were harvested by handpicking method. Because of their shorter shelf life after sorting and cleaning, fruits were kept in a refrigerator until they were utilized. All the chemicals were of analytical grade and purchased from Merck.

### 2.2 Preparation of jam

For the preparation of jam, the collected fruits (karonda and apple) were washed and cut in half. The water and fruit were added to a saucepan. The fruit was cooked for 10–20 min until it was soft. Then, the fruit was mashed thoroughly and strained through a muslin cloth using a spoon to extract the pulp. The resulting juice had a pink color.

**FIGURE 2:**
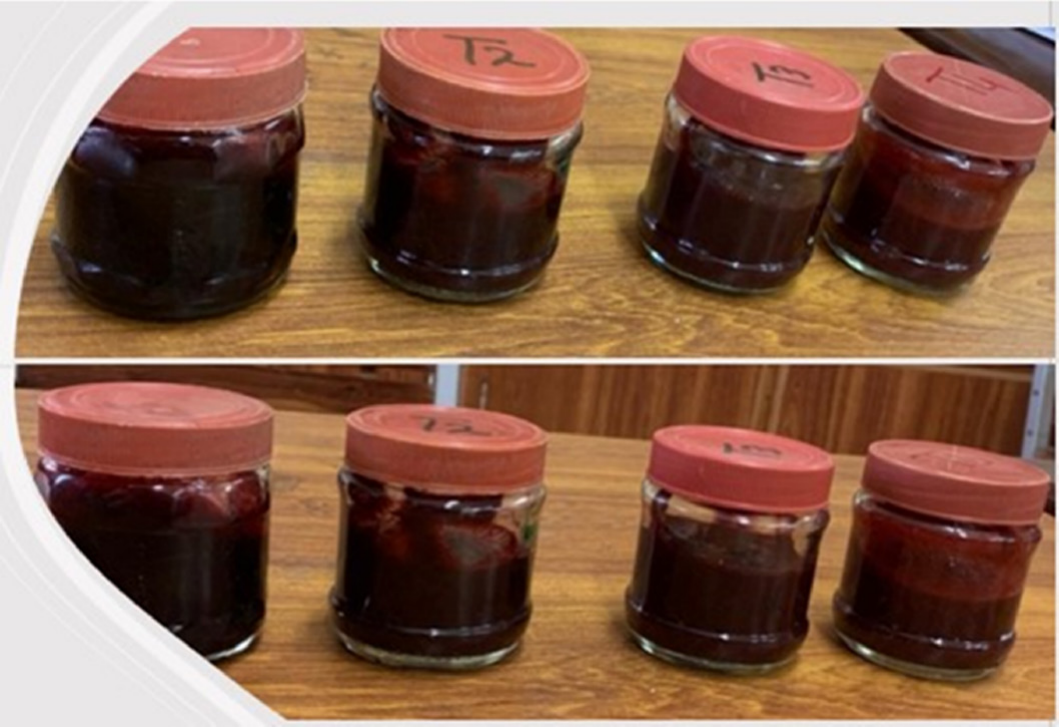
Karonda and apple jam prepared in the laboratory.

The pulp and sugar were added to a saucepan, and the concentration of sugar was determined through a drop test, TSS (68% to 70%), and sheet test to reach the endpoint. Subsequently, the jam was poured into respective glass jars for each treatment, following the method of Shahnawaz and Shiekh (2011). In the laboratory, four treatments of karonda and apple jam were prepared using different concentrations of karonda fruit and apples (Figure 2). The treatments were as follows: T1 (100% karonda + 0% apple), T2 (75% karonda + 25% apple), T3 (50% karonda + 50% apple), and T4 (25% karonda + 75% apple) (Table 1).

**TABLE 1:** Treatments of karonda and apple jam.

Treatments	Karonda pulp	Apple pulp
T_1_	100%	0%
T_2_	75%	25%
T_3_	50%	50%
T_4_	25%	75%

The karonda jam was stored in a refrigerator at 4°C for a storage period of 4 months. All samples were subsequently analyzed for physicochemical characteristics, including titratable acidity, pH, moisture content, ash content, total phenolic content, antioxidant activity, ascorbic acid, and antioxidants. Additionally, sensory characteristics such as color, taste, texture, and overall acceptability were evaluated at zero day and after 120 days.

### 2.3 Methods of analysis

#### 2.3.1 pH

The pH of the jam and fruit samples was measured using a pH meter (Bench‐top pH meter KDD002 AUXI Lab, Spain), following the AOAC guidelines (2006), 981.12 method. Prior to taking the readings, the pH meter (Bench‐top pH meter KDD002 AUXI Lab, Spain) was calibrated using pH 4.0 and 9.0 buffers. The electrode was rinsed and dried before collecting data for each replication.

#### 2.3.2 Titratable acidity (TA) (%)

The titratable acidity (TA) of both fruit samples and jam treatments was measured following the guidelines of the Association of Official Analytical Chemists (AOAC, 2006), as reported by Soares et al. (2022). For this, 5 g of fruit pulp/jam was taken from randomly selected fruits, homogenized, and mixed with 20 mL of purified water. The mixture was then filtered to obtain a clear extract. A 5 mL aliquot of the extract was titrated with 0.1N NaOH, using a few drops of phenolphthalein as an indicator, until a light pink color appeared as the endpoint. The process was replicated three times, and the data were recorded. Titratable acidity was calculated in terms of ascorbic acid using the following formula:

$$\mathrm{TA}\%=\frac{\mathrm{mL}\;\text{of NaOH used}\times \text{normality of NaOH}\times \text{equiv}.\;\text{weight of NaOH}}{\text{Weight of sample}\times \text{Volume of aliquot}}\times 100$$

#### 2.3.3 Total soluble solids

The total soluble solids (TSS) were determined following the guidelines of the Association of Official Analytical Chemists (AOAC, 2006; Method No. 960.20). A digital handheld refractometer (Atago PAL‐1) was used to measure the TSS at room temperature. For each sample (product), one drop of the extracted juice was placed on the dry refractometer's prism, and the readings were recorded in °Brix.

#### 2.3.4 Moisture content

The moisture content of karonda fruit was analyzed using the standard method outlined by the Association of Official Analytical Chemists (AOAC, 2006; ISO712:1998) employing a gravimetric principle. Watch glass dishes were selected and dried in an oven at 105°C for 20 min. Once dried, they were transferred to a desiccator, allowed to cool for 5 min, and weighed. Around 3 g of the prepared karonda fruit/jam sample was carefully transferred to the dried and weighed dishes. The dishes, along with their contents, were placed in an oven and dried for 12 h at 85°C. Following drying, the dishes and their contents were cooled in desiccators to reach room temperature for a duration of 15 min, after which they were reweighed. The loss in weight after drying the sample to a constant weight was considered as the amount of water present in the sample:

$$\text{Moisture}\%=\frac{\left(W3-W2\right)}{\left(W1-W2\right)}\times 100$$

where *W*2 is the weight of empty dishes, *W*1 is the weight of the sample, and *W*3 is the weight of the dried sample and dishes.

#### 2.3.5 Ash content

The ash content of the fruit and jam samples was determined by using the modified method of AOAC (2006; Method No. 923.03) as described by Soares et al. (2022). About 3 g of dried karonda/jam samples were transferred to the crucibles. The crucibles were placed in a muffle furnace for 2 h at 600°C and then removed from the muffle and placed in desiccators for 15 min to cool. The weight of total ash was calculated by difference and expressed as a percentage of the fresh fruit/jam sample:

$$\mathrm{Ash}\%=\frac{\left(W3-W2\right)}{\left(W1-W2\right)}\times 100$$

where *W*2 represents the weight of empty dishes, *W*1 represents the weight of the sample, and *W*3 represents the weight of the ashed sample and dishes, respectively.

#### 2.3.6 Total sugars

According to the method as reported in AOAC (2006; Method. 925.45), the reducing and non‐reducing sugar content was estimated in the karonda fruit. Total sugars were obtained by the addition of both reducing and non‐reducing sugars in the fruit sample.

#### 2.3.7 Reducing sugars

Reducing sugars were analyzed following the method outlined in AOAC (2006; Method No. 939.03). Initially, 10 mL of fruit extract was added to a cylinder, and the volume was adjusted to 100 mL by adding distilled water. This solution was subsequently transferred to a burette. In a conical flask, 5 mL of Fehling A, 5 mL of Fehling B, and 10 mL of distilled water were combined. The solution in the flask was then heated until it reached boiling point and titrated against the sample solution in the burette. The appearance of a brick‐red color indicated the endpoint, and methylene blue was added to confirm whether the color changed to blue or not. The calculation of reducing sugars was carried out using the following formula:

5mLof FehlingA+5mLof FehlingBwill reduce0.05gof reducing sugar.

5mLof FehlingA+5mLof FehlingB=XmLof10%solution=0.05gof reducing sugar.

100mLof10%solution containing

$$\frac{0.05\times 100}{X\;\mathrm{mL}}=Y\;g\;\text{of reduced sugar}$$

%reducing sugar in sample=Y×100/10.

#### 2.3.8 Non‐reducing sugars

Non‐reducing sugars were estimated using the method reported in AOAC (2006; Method No. 939.03). A 10 mL sample of fruit extract was taken in a volumetric flask, and distilled water was added to achieve a volume of 100 mL. In a separate flask, 20 mL of the diluted solution was combined with 10 mL of 1 N HCl, and the mixture was heated on a burner for 5–10 min. After heating, the sample was cooled by adding 10 mL of 1 N NaOH. The resulting sample solution was then transferred to a burette. In a conical flask, 5 mL of Fehling A, 5 mL of Fehling B, and 10 mL of distilled water were mixed and heated until boiling. The solution in the flask was titrated against the sample in the burette until a brick‐red color appeared, indicating the endpoint. The presence of the brick‐red color was confirmed by adding methylene blue as an indicator. Non‐reducing sugars were calculated using the following formula:

Non−reducing sugar=Total reducing sugar−Free reducing sugar.

#### 2.3.9 Antibacterial activity

Karonda fruit extract was tested for its antimicrobial activity using the agar well plate method. This method was adapted from a previously published study by Doshi et al. (2017) with some modifications. The aim was to determine the antibacterial potential of the karonda fruit extract against two bacterial strains, namely *Bacillus subtilis* and *Stenotrophomonas pavinii*. Luria bertani agar base medium was used as the growth medium. To begin, the agar plates were streaked with fresh bacterial suspensions of *B. subtilis* and *S. pavinii*. The plates were then incubated at 37°C for 24 h to allow the bacterial cultures to grow. After the incubation period, wells were created on the agar plates using a sterile pipette tip. Subsequently, 50 μL of karonda fruit extract was poured into the wells. As a control, a sterile paper disc was also placed on the inoculated plates. The antibacterial activity was observed by examining the clear zones of inhibition around the wells. The zones of inhibition indicated the extent of inhibition of bacterial growth caused by the karonda fruit extract.

#### 2.3.10 Mineral analysis of the fresh and dried karonda fruits

Karonda fruit was analyzed for iron, calcium, manganese, zinc, and potassium via Atomic Absorption Spectrophotometer (AA‐7800 Shimadzu, Japan) following the previously adopted method by Herrera et al. (2023). For quantification of metals, the calibration curves were prepared with standard solutions of calcium, iron, manganese, zinc, and potassium.

### 2.4 Determination of phytochemicals

To determine the anthocyanin, total phenolic content, antioxidant, and flavonoid levels in each fresh, sun‐dried fruit, and jam sample, a 5‐g portion of each sample was soaked in 100 mL of a 40% ethanol‐water solution for 24 h. The resulting extracts were then filtered using No.4 Whatman filter papers and utilized for subsequent analysis.

#### 2.4.1 Ascorbic acid (vitamin C) content (mg/100 g)

The vitamin C content of both fruit and jam samples was measured using the method described in AOAC (2006; Method No. 967.22). The determination of vitamin C content involved the utilization of dye (2,6‐dichlorophenol indophenol). Firstly, a 5 mL sample (product) was placed in a 100 mL conical flask. Then, 5 mL of a 4% meta‐phosphoric acid solution was added to the flask. The sample was subsequently titrated with the 2,6‐dichlorophenol indophenol dye until a light pink color indicated the endpoint. The vitamin C content was estimated using the following formula:

$$\text{Ascorbic acid}\;\left(\mathrm{mg}/10\text{0g}\right)=F\times T\times 100/S\times D$$

where *F* represents the standardization factor, which is the ratio of milliliters of ascorbic acid to milliliters of pigment used. *T* represents the milliliters of pigment used for the sample. *S* represents the milliliters of a diluted sample taken for titration, and *D* represents the milliliters of the sample taken for dilution.

#### 2.4.2 Total anthocyanin

The total anthocyanin content was measured using a spectrophotometer (UV‐400 spectrophotometer, Hamburg, Germany) and the pH dilution method described by Golmohamadi et al. (2013). Two dilutions of the fruit and jam samples were prepared using potassium chloride buffer (pH 1.0) (0.5 mL sample extracts and 3.5 mL potassium chloride) and sodium acetate buffer (pH 4.5) (0.5 mL sample extract and 3.5 mL sodium acetate) against a blank, followed by a 15‐min equilibration period. The absorbance of each dilution was measured at 515 nm and 700 nm using a UV–Vis spectrophotometer (UV‐400 spectrophotometer, Hamburg, Germany). The anthocyanin pigment was calculated as milligrams of cyanidin‐3‐glucoside per liter using an extinction coefficient of 29,600 and a molecular weight of 449.2.

#### 2.4.3 Antioxidant activity

The antioxidant activity was evaluated using the DPPH method as reported by Williams et al. (1995). To prepare the extracts, different concentrations (1000 μg/mg) of 0.5 mL from each fruit sample and jam treatment were taken. Then, 1 mL of freshly prepared DPPH (0.25 mM) and 1 mL of ethanol were added to each extract. The samples were thoroughly mixed and kept in the dark at room temperature for 30 min. Afterward, the DPPH radical scavenging activity of each sample was measured using a spectrophotometer (UV‐400 spectrophotometer, Hamburg, Germany) at 517 nm. The antioxidant activity was calculated by using the following formula:

$$\text{DPPH scavenging activity}\%=\left(A_{0}-A_{S}/A_{0}\right)\times 100$$

where *A*_0_ is the absorbance of the control and *A*_S_ is the absorbance of the sample.

#### 2.4.4 Total phenolic content

The total phenolic content of both the fruit and jam samples was estimated using a spectrophotometric‐modified method as described by Singh et al. (2017) and Jankulovska et al. (2023). To perform the evaluation, 2.5 mL of 10% Folin‐Ciocalteau's Reagent and 2 mL of 7.5% sodium carbonate solution were combined, followed by the addition of a 0.5 mL sample. The sample was then incubated at 45°C for 40 min, and the absorbance was measured using a spectrophotometer at a wavelength of 765 nm. The average value of the total phenolic content was determined by calculating the mean of three readings.

#### 2.4.5 Total flavonoids

The total flavonoid content of the fruit and jam product was determined using a method previously reported by Chang et al. (2002). A sample weighing 0.1 g was mixed with 0.1 M potassium acetate, followed by the addition of 0.1 mL of aluminum chloride and 2.8 mL of distilled water. The resulting solution was mixed thoroughly and incubated for 30 min at room temperature. The absorbance of the reaction mixture was then measured using a UV–Vis spectrophotometer at a wavelength of 430 nm, with a blank used for reference. The results were calculated using the quercetin curve.

### 2.5 Sensory evaluation

An organoleptic test was conducted to evaluate sensory parameters including color, taste, texture, and overall acceptability, using a panel of ten judges. The differences between samples were assessed using a 9‐point hedonic scale, ranging from “extremely liked” (9) to “extremely disliked” (0), following the requirements of ISO 6658 as previously mentioned by Banav et al. (2018).

### 2.6 Statistical analysis

The collected data were analyzed both statistically and graphically. Analysis of variance (ANOVA) was conducted to assess the means of the physicochemical, nutritional, and sensory evaluations using Origin Pro 8 software.

## 3 RESULTS

### 3.1 Physicochemical analysis of karonda fruit

**FIGURE 3:**
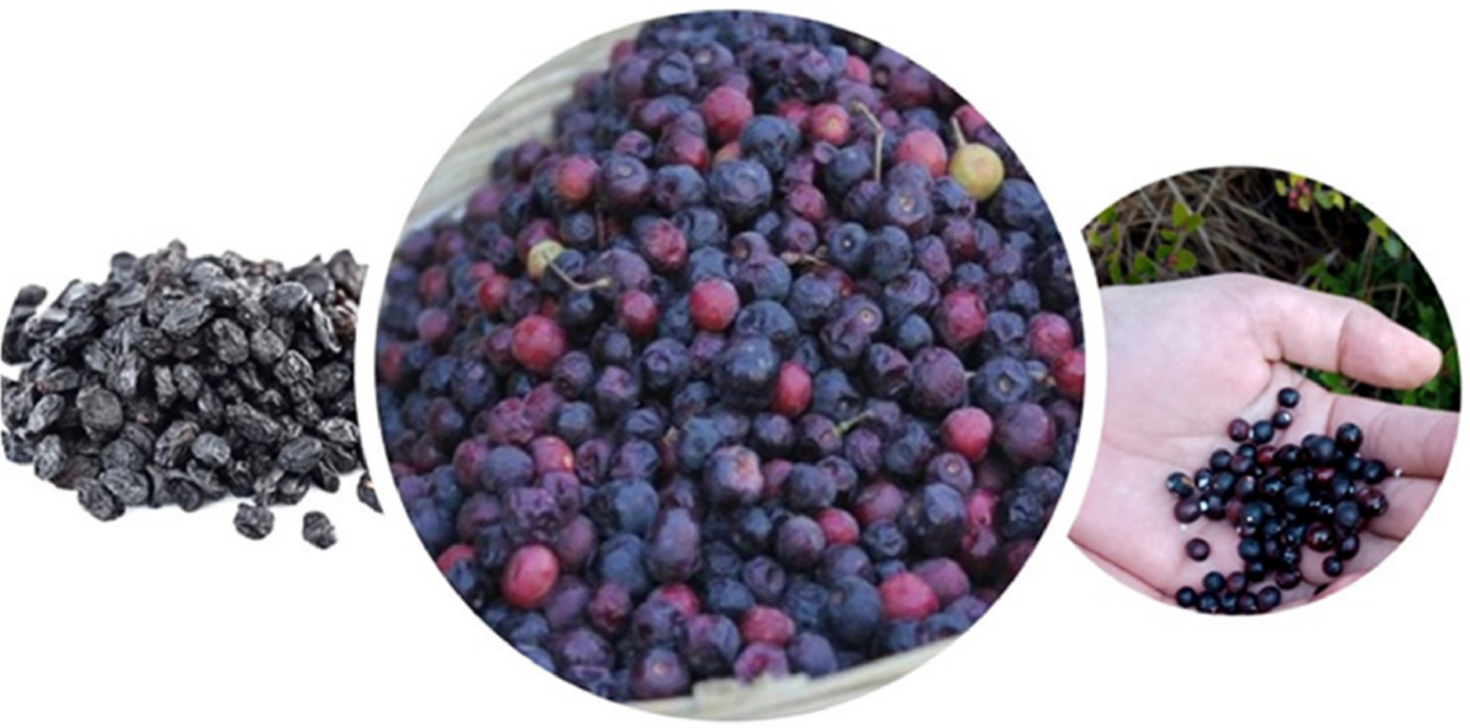
Dry and fresh karonda fruit samples.

The results of the analysis of the physicochemical parameters of both dry and fresh karonda fruits are presented in Table 2. The moisture content of the dry karonda fruit sample was determined to be 36.00%, which is significantly lower than the moisture content of the fresh karonda fruit sample, which was found to be 66.50% (Figure 3). Furthermore, the crude fat content of the dry karonda fruit was measured at 19.00%, which is significantly higher than the crude fat content of the fresh karonda fruit, which was found to be 2.570%. Similarly, the crude fiber content of the dry karonda fruit was determined to be 4.90%, which is significantly higher than the crude fiber content of the fresh karonda fruit, which was found to be 1.01%. The analysis revealed that the ash content of the dry karonda fruit was determined to be 4.9850%, which is significantly higher than the ash content of the fresh karonda fruit, which measured at 1.81%. Furthermore, the pH of the dry karonda fruit was found to be 8.1, significantly higher than the pH of the fresh karonda fruit, which was measured at 6.70. In terms of total soluble solids, the dry karonda fruit exhibited a value of 5.60%, which was significantly higher than the 1.81% total soluble solids of the fresh karonda fruit. Moreover, the total acidity of the dry karonda fruit was measured at 0.450%, significantly lower than the total acidity of the fresh karonda fruit, which was determined to be 1.870%. Lastly, the vitamin C content of the dry karonda fruit was found to be 49.00 mg/100 g, significantly lower than the 72.00 mg/100 g vitamin C content of the fresh karonda fruit. The total phenolic content of the dry karonda fruit was determined to be 52.50 mg GAE/g, which is significantly higher than the total phenolic content of the fresh karonda fruit, which was 17.20 mg GAE/g. The reducing sugars content of the dry karonda fruit was found to be 2.900%, which is significantly lower than the reducing sugars content of the fresh karonda fruit, which measured at 2.300%. Additionally, the non‐reducing sugars content of the dry karonda fruit was determined to be 5.150%, significantly higher than the non‐reducing sugars content of the fresh karonda fruit, which was found to be 3.60%. Furthermore, the total sugars content of the dry karonda fruit was measured at 8.05%, significantly higher than the total sugars content of the fresh karonda fruit, which was determined to be 5.90%. The antioxidant activity of the dry karonda fruit was found to be 0.180%, which is significantly higher than the 0.0249% antioxidant activity of the fresh karonda fruit due to increased anthocyanin content of dried karonda fruit. Moreover, the flavonoid content of the dry karonda fruit was found to be 1.4450%, significantly lower than the flavonoid content of the fresh karonda fruit, which was determined to be 0.8550%. Additionally, the anthocyanin content of the dry karonda fruit was determined to be 50.095%, significantly higher than the anthocyanin content of the fresh karonda fruit, which measured at 12.185%.

**TABLE 2:** Chemical analysis of karonda fruit.

Physiochemical parameters	Dry karonda fruit	Fresh karonda fruit
Moisture	36.000 ± 1.414**	66.500 ± 0.007**
Crude fat	19.000 ± 1.414**	2.570 ± 0.014
Crude fiber	4.9050 ± 0.077**	1.0100 ± 0.028
Ash %	4.9850 ± 0.021**	1.8100 ± 0.070
pH	8.1000 ± 0.141**	6.7000 ± 00
Total soluble solids	5.6000 ± 0.565**	1.81 ± 0.070
Total acidity %	0.4500 ± 0.071**	1.8700 ± 0.028
Vitamin C mg/100 g	49.000 ± 0.071	72.000 ± 1.414**
Total phenolic content mgGAE/g	52.500 ± 0.707**	17.200 ± 0.282**
Reducing sugars %	2.9000 ± 0.141**	2.3000 ± 0.141
Non‐reducing sugars %	5.1500 ± 0.212**	3.6000 ± 0.141
Total sugars %	8.0500 ± 0.354**	5.9000 ± 00
Antioxidant activity %	0.1800 ± 0.014**	0.0249 ± 0.000
Flavonoid content	1.4450 ± 0.007**	0.8550 ± 0.035
Anthocyanin content	50.095 ± 1.280**	12.185 ± 0.001
Carbohydrates	35.000 ± 0.099**	28.165 ± 1.803
Energy (J)	43.500 ± 3.536**	17.500 ± 00

*Note*: Different numbers of * shows significant differences.

Furthermore, the carbohydrates content of the dry karonda fruit was found to be 35.000%, significantly higher than the carbohydrates content of the fresh karonda fruit, which was measured at 28.165%. Lastly, the energy content of the dry karonda fruit was determined to be 43.500 joules, significantly higher than the energy content of the fresh karonda fruit, which measured at 17.500 joules.

### 3.2 Mineral analysis

The iron content of the fresh karonda fruit was found to be 42%, which is significantly higher than the iron content of the dry karonda fruit, which remained at 3.14%. Additionally, the calcium content of the fresh karonda fruit was estimated to be 24%, significantly higher than the calcium content of the dry karonda fruit, which was found to be 2.680% (Table 3). Moreover, the manganese content of the dry karonda fruit was measured at 257.5 mg/kg, significantly lower than the manganese content of the fresh karonda fruit, which was measured at 533.5 mg/kg. Similarly, the zinc content of the dry karonda fruit was found to be 133.5 mg/kg, significantly lower than the zinc content of the fresh karonda fruit, which was measured at 413.6 mg/kg. Furthermore, the potassium content of the dry karonda fruit was determined to be 1.950%, significantly lower than the potassium content of the fresh karonda fruit, which measured at 79.13% (Table 3). The protein content was not detected in either the fresh or dried fruit. However, the total CO_2_, energy, total phenol, total flavonoids, total sugar, reducing sugar, non‐reducing sugar, anthocyanin, and antioxidant content were significantly higher in the dried fruit compared to the fresh fruit. In summary, the drying process results in a reduction in moisture content, pH, and total acidity, while simultaneously increasing the levels of crude fat, ash, fiber, and total soluble solid content. Furthermore, it leads to a decrease in vitamin C content while enhancing certain nutritional and antioxidant properties of karonda fruit.

**TABLE 3:** Mineral analysis of karonda fruit.

Mineral content (mg/kg)	Dry karonda fruit	Fresh karonda fruit
Iron	3.14	42*
Calcium	2.680	24**
Manganese	257.5	533.5**
Zinc	133.5	413.6**
Potassium	1.950	79.13**

*Note*: Different numbers of * shows significant differences.

### 3.3 Antimicrobial effect of karonda fruit

**FIGURE 4:**
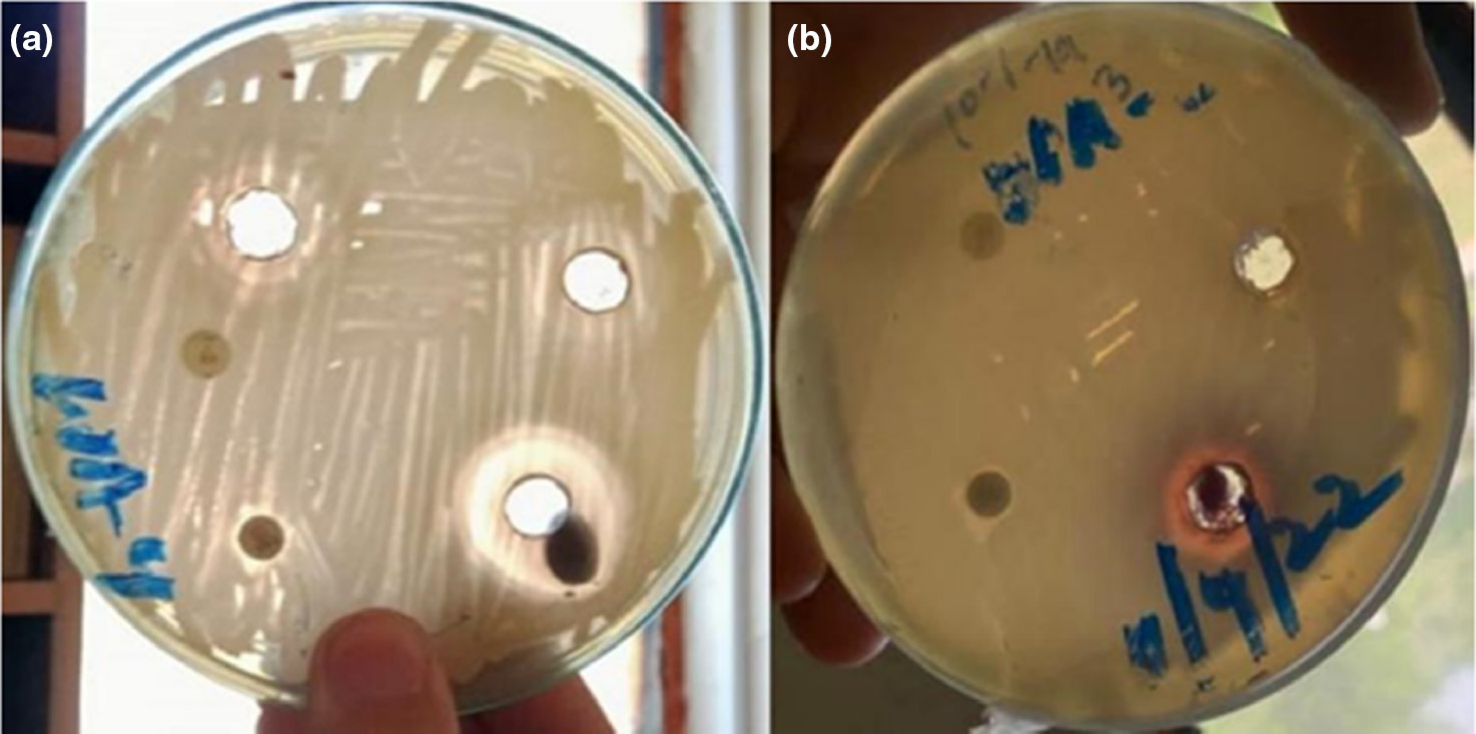
Antimicrobial effect of karonda fruit (a) *Stenotrophomonss pavinii* and (b) *Bacillus subtilis*.

The berries of karonda exhibited antimicrobial properties when tested against a nonpathogenic strain of *Bacillus subtilis* and a pathogenic bacterial isolate of *Stenotrophomonas pavinii* using a well plate assay (Figure 4). The bactericidal properties of the berries can be attributed to the presence of bioactive compounds in the form of phenolics and flavonoids. Our findings are supported by the previous report of Doshi et al. (2017), who demonstrated the antibacterial activity of Nano formulations prepared from the fruits of *C. carandas* and *C. spinarum* against *Staphylococcus aureus*, *Bacillus subtilis*, and *Salmonella typhi*. Additionally, they reported that karonda possessed excellent antibacterial activity against *Escherichia coli*. Previously, several studies have reported the antimicrobial properties of cranberries, as documented by Caillet et al. (2012), and in four cultivars of blueberries, as explored by Shen et al. (2014). The researchers have also suggested that the presence of phytochemicals in berries influences the microbial communities.

### 3.4 Physicochemical analysis of jam

Tables 4 and 5 present the results of the studied parameters of karonda fruit jam during storage. The experiment consisted of four different treatments (T1‐T4), and the measured parameters included pH, acidity, moisture content, ash content, ascorbic acid content, total acidity (TA), total phenolic content, total flavonoid content, and antioxidant activity. Measurements were taken at day zero and after 120 days of storage.

**TABLE 4:** Chemical parameters of karonda jam at zero day of storage.

Treatment's	pH day at zero day of storage	Acidity(%) at zero day of storage	Moisture(%) at zero day of storage	Ash(%) at zero day of storage	Ascorbic acid (mg/100 g) at zero day storage	Total anthocyanin (mg GAE/g) at zero day of storage	Total phenolic (mg GAE/g) at zero day of storage	Total flavonoid (mg GAE/g) at zero day of storage	Antioxidant activity (%) at zero day of storage
T1	4.0 ± 0.078	14 ± 1.556	28 ± 4.243	15 ± 1.414	72 ± 2.828	161.97 ± 1.416	20.56 ± 1.571	1.03 ± 0.014	0.01635 ± 0.016
T2	4.70 ± 0.014	9.8 ± 1.414	35 ± 1.414	13 ± 2.828	36 ± 1.414	151.96 ± 1.428	19.62 ± 1.571	1.10 ± 0.014	0.014 ± 0.001
T3	4.60 ± 0.014	8.4 ± 2.263	25 ± 4.243	11.6 ± 1.598	32.4 ± 1.131	75.97 ± 1.415	22.93 ± 1.508	1.12 ± 0.014	0.0078 ± 0.002
T4	4.48 ± 0.156	8.4 ± 1.131	37 ± 2.828	7.28 ± 1.414	18 ± 1.414	41.74 ± 1.428	13.56 ± 1.570	1.03 ± 0.014	0.0261 ± 0.016

**TABLE 5:** Chemical parameters of karonda jam after 4 months (120 days) of storage.

Treatments	pH	Acidity (%)	Moisture (%)	Ash (%)	Ascorbic acid (mg/100 g)	Total anthocyanin (mg GAE/g)	Total phenolics (mg GAE/g)	Total flavonoid (mg GAE/g)	Antioxidant (%) activity
T1	3.27 ± 0.156	16.8 ± 0.00	43.6 ± 1.556	14.2 ± 1.570	52.2 ± 2.546	175.33 ± 1.571	59.325 ± 0.160	0.86 ± 0.071	0.00555 ± 0.000
T2	3.95 ± 0.014	14 ± 1.980	46.3 ± 1.570	10.5 ± 1.556	34.2 ± 2.546	32.562 ± 1.571	47.686 ± 1.598	0.71 ± 0.007	0.00725 ± 0.018
T3	4.02 ± 0.014	12.6 ± 0.00	50 ± 1.414	8.11 ± 1.428	33 ± 2.828	49.216 ± 1.571	39.211 ± 2.077	0.76 ± 0.028	0.0073 ± 0.000
T4	4.01 ± 0.014	13.6 ± 0.00	58 ± 0.00	5.5 ± 0.00	15 ± 0.00	19 ± 0.00	13 ± 0.00	0.45 ± 0.00	0.002 ± 0.00

*Note*: Four treatments: T1 = 100% karonda pulp + 0% apple pulp, T2 = 75% karonda pulp + 25% apple pulp, T3 = 50% karonda pulp + 50% apple pulp, T4 = 25% karonda pulp + 75% apple pulp.

#### 3.4.1 Treatment T1

Treatment T1 used 100% karonda pulp and 0% apple pulp. It had a pH of 4.0 on the first day, which decreased to 3.27 after 120 days of storage. The acidity increased from 14% on the first day to 16.8% after 120 days. The moisture content rose from 28% on the first day to 43.6% after 120 days. The ash content was 15% initially, but it decreased to 14.2% after 120 days. The ascorbic acid content started at 72% on the first day and decreased to 52.2% after 120 days. The total acidity was 14% ± 1.556 on the first day, and it increased to 16.8% ± 0.00 after 120 days. The total phenol content was 20.56 mg GAE/100 g initially and increased to 59.325 mg GAE/100 g after 120 days. The total flavonoid content was 1.03 mg QE/100 g on the first day, but it decreased to 0.86 mg QE/100 g after 120 days. The antioxidant activity started at 0.0276 on the first day and decreased to 0.0055 after 120 days.

#### 3.4.2 Treatment T2

The treatment T2 consisted of 75% karonda pulp and 25% apple pulp. It exhibited a pH of 4.7 on the first day, which decreased to 3.95 after 120 days of storage. The initial acidity was 9.8%, which rose to 14% after 120 days. The moisture content started at 35% and increased to 46.3% after 120 days. The ash content was 13% initially but decreased to 10.5% after 120 days. Ascorbic acid content began at 36% and decreased to 34.2% after 120 days. The total acidity was 9.8% on day one and increased to 14% after 120 days. Total phenol content was 19.62 mg GAE/100 g initially and rose to 47.686 mg GAE/100 g after 120 days. The total flavonoid content started at 1.1 mg QE/100 g and decreased to 0.71 mg QE/100 g after 120 days. Antioxidant activity was 0.014 on the first day and increased to 0.02013 after 120 days.

#### 3.4.3 Treatment T3

Treatment 3 comprised a combination of 50% karonda pulp and 50% apple pulp. Its pH level was 4.6 on the initial day and decreased to 4.02 after a 120‐day storage period. The acidity started at 8.4% on day one and rose to 12.6% after 120 days. Moisture content began at 25% and increased to 50% over the same period. The ash content initially measured 11.6% but was reduced to 8.11% after 120 days. Ascorbic acid content stood at 32.4% initially and decreased slightly to 33% after 120 days. Total acidity on day one was 8.4% and decreased to 12.6% after 120 days. The total phenol content started at 22.93 mg GAE/100 g and declined to 39.211 mg GAE/100 g after 120 days. The total flavonoid content was 1.12 mg QE/100 g initially and decreased to 0.76 mg QE/100 g after 120 days. Finally, the antioxidant activity measured 0.0078 on the first day and decreased to 0.0073 after 120 days.

#### 3.4.4 Treatment T4

Treatment T4 utilized a mixture of 25% karonda pulp and 75% apple pulp, resulting in a pH value of 4.48, acidity of 8.4%, moisture content of 37%, ash content of 7.28%, ascorbic acid content of 18%, total anthocyanin content of 41.74%, total phenol content of 13.56%, total flavonoid content of 1.03%, and total antioxidant activity of 0.02615 on the first day. However, after 120 days of storage, treatment T4 (75% apple and 25% karonda) experienced spoilage. This can be attributed to apples being more susceptible to spoilage compared to karonda fruit due to their higher water content, which promotes microbial growth and oxidation.

## 4 DISCUSSION

### 4.1 Physicochemical properties of karonda fruit

The results suggest that the drying process enhances the antioxidant activity of karonda fruit, which has been reported in previous studies by Jain et al. (2016) and Bhati and Goyal (2019). This increase in antioxidant activity can be attributed to the rise in phenolic and flavonoid compounds. Additionally, the results demonstrate that the drying process significantly reduces the vitamin C content of the fruit. Vitamin C, also known as ascorbic acid, is a water‐soluble vitamin. Fresh karonda fruit contains 2.570% fats, 1.810% ash, and 28.16% carbohydrates, and these results are in agreement with the findings of Siyuma and Meresa (2021) who reported almost similar value in *C. spinarum* collected from two different sites in Ethiopia. However, in dried karonda fruit, 19.00% fats, 4.985% ash, and 35.00% carbohydrates were found. Similar findings have been reported in goji berries by Niro et al. (2017) and Endes et al. (2015). The high‐fat content of karonda fruit compared to other berries is due to the presence of edible seeds.

In terms of pH, the results of this study demonstrate that fresh karonda fruit has a pH of 8.1, which is quite higher than the previously reported study of Siyuma and Meresa (2021), and this might be due to the environmental and growing conditions of soil in two different countries. Regarding moisture content, the findings of this study reveal that fresh karonda fruit contains 67% moisture which is little higher than the findings of Siyuma and Meresa (2021) while dried karonda fruit contains 36%. The moisture content is higher than that of other dried berries like cranberries (8.5%–14.9%) (Naczk & Shahidi, 2004) and blueberries (5%–10%) (Cao et al., 2006), but lower than that of blackberries (80%–90%) (Garcia‐Salguero et al., 2017). The lowest amount of inverted sugar (5.900%) was found in the fresh fruit. During the drying process, the content of inverted sugar increased, reaching a value of 8.050%. Since the dried fruit has less water content, it concentrates all the sugar and calories into much smaller packages. Consequently, fresh karonda fruit contains fewer calories (17.500 kcal of energy), whereas dried fruit contains significantly higher calories (43.500 kcal of energy) and sugar, including both reducing and non‐reducing sugars. The obtained results, in accordance with the available literature data, fall within the range of 6.20% to 10.80% (Konic‐Ristic et al., 2013).

For fiber content, the fresh fruit exhibited the lowest level (1.010%), while the dry fruit had the highest level (4.905%). The fiber content of various berry fruits ranges from 1.66% to 5.90%. The distribution of fiber content in karonda fruit is similar to the crude fiber content found in other wild berries, such as Crataegus monogyna berries and strawberries, as mentioned by Souci et al. (2008). Our data suggest that dry karonda fruit contains a significant amount of dietary fiber, and the recommended daily intake of dietary fiber for adults is 25 g/day, according to the report of LARN (2014). Therefore, incorporating this berry fruit into the diet as spices or in the form of its valuable products can enhance the dietary fiber level, which is beneficial for overall human health. In fresh karonda fruit, the observed value of Total Soluble Solids (TSS) is 1.81%, whereas in dried fruit, the TSS value is 5.600%. The high TSS value in dry karonda fruit can be attributed to the significant reduction in moisture content and the subsequent increase in TSS concentration.

Regarding anthocyanin content, the results of this study indicate that fresh karonda fruit has an anthocyanin content of 50.095 mg/L, which falls within the range observed in other berries, such as blueberries (25.41–106.38 mg/L) as reported by Ljubica and Frosina (2015), and blackberries (14.28–35.89 mg/L) from the study conducted by Garcia‐Salguero et al. (2017). In contrast, dry fruit exhibits the lowest anthocyanin content (12.18 mg/L) due to the instability of anthocyanin under light, pH, oxygen, temperature changes, and enzymatic activity. Consequently, the drying process, which involves temperature and oxygen variations, leads to a reduction in the anthocyanin content of the dried fruit. The literature supports a wide range of anthocyanin content in berries, spanning from 25.41 to 106.38 mg/L, as reported by Ljubica and Frosina.

The highest observed phenolic content was found in fresh fruit (52.500 mg GAE/g), whereas the lowest content was observed in dry fruit (17.200 mg GAE/g). During the sun drying process, there was a significant decrease in total polyphenolic content, with the largest percentage decrease. A similar decreasing trend was also observed in bilberries, as reported by Michalczyk et al. (2009). The antioxidant activity and total flavonoid content were lowest in fresh fruit (0.0249% and 0.8550, respectively), while the highest antioxidant activity and total flavonoid content were measured in dry fruit (0.1800% and 1.4450, respectively). Dry fruits contain a significant amount of fiber and serve as a great source of antioxidants, which is why they contain higher levels of antioxidants compared to fresh fruits. The obtained data align with previous literature reports indicating that antioxidant activity and flavonoid content increase after drying, as reported by Sona et al. (2015).

In the dry fruit of karonda, the observed mineral content included iron (3.14%), calcium (2.680%), manganese (257.5 mg/L), zinc (133.5 mg/L), and potassium (1.950%). On the other hand, fresh karonda fruit exhibited 24% calcium and 42% iron content. These findings align with those of Banik et al. (2011), who reported similar mineral observations in karonda fruit.

The analysis results of both dry and fresh karonda (*Carissa carandas*) fruits align with findings from other studies conducted on berries. For instance, Wang et al. (2019) conducted a study that demonstrated how blueberries possess higher moisture, crude fat, and ash content compared to other berries like strawberries, raspberries, and blackberries. The higher water content in blueberries makes them more susceptible to oxidation and microbial spoilage. The study also revealed that blueberries exhibited elevated levels of vitamin C, total phenolics, and antioxidant activity compared to other berries. Additionally, blueberries showcased higher quantities of reducing sugars, non‐reducing sugars, total sugars, and flavonoids than other berries. This is likely due to the presence of anthocyanins, responsible for the blue color of blueberries and contributing to their antioxidant properties.

Likewise, Bhatia et al. (2018) conducted a study that found blackberries to have higher levels of moisture, crude fat, crude fiber, ash, total soluble solids, total acidity, vitamin C, total phenolics, reducing sugars, non‐reducing sugars, total sugars, flavonoids, and anthocyanins compared to other berries such as raspberries, strawberries, and blueberries. These results suggest that karonda fruit exhibits a composition similar to that of other berries, thus making it a valuable addition to a healthy diet.

### 4.2 Jam

All the treatments in the present experiment have a significant impact on all observed traits. However, the treatments varied greatly from each other at different time points, as demonstrated in Tables 5 and 6. The results indicate that an increase in the proportion of apple pulp in the jam leads to a decrease in the pH of the jam and the content of ascorbic acid. This is likely due to the fact that apple pulp has a lower pH and higher acidity compared to karonda pulp, which can consequently lower the overall pH and acidity of the jam, as mentioned by Biswas et al. (2011). Additionally, apples are not as rich in vitamin C as karonda, resulting in a decrease in the ascorbic acid content of the jam.

**TABLE 6:** Sensory parameters (storage period marks according to 9‐point Hedonic scale) of karonda jam during storage.

Treatment's	Color of jam after storage (1–9)		Flavor of jam after storage(1–9)		Taste of jam after storage(1–9)		Overall acceptability of jam after storage(1–9)	
	0	120	0	120	0	120	0	120
T1	8	7	8	7	6	5	7	5
T2	9	8	7	6	7	6	7	6
T3	9	7	9	8	9	8	9	8
T4	9	7	9	7	9	7	9	7

The decrease in pH, ascorbic acid content, total phenol content, and antioxidant activity observed over time during the storage of karonda fruit jam, as shown in Tables 4 and 5, is likely attributable to a combination of chemical and physical changes that occur in fruits and berries during storage. This is supported by the study conducted by Guo et al. (2018), which determined that the decline in the pH of strawberry jam was caused by the release of organic acids (such as citric acid) from the fruit. The study concluded that there was a positive correlation between the acidity of the jam and the decrease in pH.

In all treatments, the ascorbic acid content experienced a decrease, with the most significant decline observed in T1 (52.2% after 120 days). This finding aligns with the study conducted by Varela et al. (2015), which also discovered a decreasing trend in the ascorbic acid content of fruits and vegetables during storage, attributed to oxidation. The study concluded that the loss of ascorbic acid was more pronounced in fruits and vegetables with high levels of ascorbic acid, such as citrus fruits and peppers, particularly with prolonged storage duration.

Total phenolics, total flavonoids, and antioxidant content decreased in all treatments, with the greatest decrease observed in T1 (59.325, 1.03, 0.0055, respectively, after 120 days). The decrease in total phenolic content and antioxidant activity over time is also likely due to oxidation. Phenolic compounds, including flavonoids and anthocyanins, play a crucial role in the antioxidant activity of fruits and berries. However, these compounds are also highly reactive and can easily undergo oxidation during storage, leading to a reduction in their content and antioxidant activity. This is supported by a study conducted by Adjimane et al. (2017), which found that the decline in total phenolic content and antioxidant activity of fruits and vegetables during storage was attributable to oxidation. The study concluded that the loss of phenolic compounds, such as flavonoids and anthocyanins, was the primary cause of the decline in antioxidant activity.

Another factor that can contribute to these changes during storage is the release of water and other compounds from the fruit. As the moisture content of the jam increases, it can affect the acidity, pH, and ascorbic acid content, resulting in the observed changes in the results. A study conducted by El‐Nemr et al. (2019) and published in the Journal of Food Processing and Preservation examined the chemical and microbiological changes in a strawberry jam during storage. The study discovered that pH, acidity, and total soluble solids decreased over time, while moisture content and microbial counts increased. Additionally, the study observed a decline in the antioxidant activity of the jam over time. These findings align with our study on karonda fruit jam, which also demonstrated a reduction in pH, ascorbic acid content, total phenol content, and antioxidant activity during storage.

Another study conducted by Yılmaz et al. (2019) and published in the Journal of Food Science and Technology examined the quality changes in blackberry jam during storage. The study revealed that pH, acidity, total soluble solids, and ascorbic acid content decreased over time, while moisture content and microbial counts increased. Additionally, the study observed a decline in the antioxidant activity of the jam over time. These findings align with our study on karonda fruit jam.

Yet another study conducted by A. S, R., S. R. K, S. V. G. and S. V. R (2018) examined the quality changes in raspberry jam during storage. The study revealed that pH, acidity, total soluble solids, and ascorbic acid content decreased over time, whereas moisture content and microbial counts increased. Furthermore, the study observed a decline in the antioxidant activity of the jam over time. These findings align with the results of our study on karonda fruit jam.

The most important and noteworthy aspect is that the treatments with high karonda fruit concentration (T1, T2, T3) remained unchanged even after 120 days of storage without preservation. This is attributed to the stable composition of karonda fruit, making it less susceptible to oxidation and other forms of degradation. Moreover, karonda fruit possesses antimicrobial properties. Additionally, the presence of sugars and acids in karonda fruit aids in reducing the respiration rate, thereby preserving the quality of the jam. On the other hand, treatment T4 (75% apple and 25% karonda) experienced spoilage after 120 days of storage. Apples are more prone to spoilage compared to karonda fruit due to their higher water content, which promotes microbial growth and oxidation. Furthermore, apples contain more enzymes, leading to quicker breakdown and spoilage compared to karonda fruit. Storage conditions also influence the spoilage of apple jam, with higher temperatures and humidity accelerating the degradation process.

#### 4.2.1 Sensory evaluation

Various sensory attributes were measured for jam such as flavor, color, taste, and overall acceptability as shown in Table 6. All the treatments are awarded better scores by the panel of 10 judges but treatments 3 and 4 are awarded the best score (as shown in Table 6); the scorecard is based on a 9‐point hedonic scale. All the treatments are showing decrease in sensory quality with the passage of storage duration which is because of blending apples with karonda as apples have less vitamin C content as well as no antimicrobial characteristics. Similar decreasing trend in sensory attributes with increasing storage intervals was observed by Banav et al. (2018) in gooseberry jam. Another study by Abdel‐Hady et al. (2014) reported decrease in the sensorial properties of strawberry jam also in agreement with our findings.

## 5 CONCLUSION

In conclusion, the results of this study demonstrate that karonda fruit holds significant potential as a source of nutrients and antioxidants. Its pH, crude fat, ash, fiber, total soluble solid content, total acidity, vitamin C, and anthocyanin content fall within the range of other berries. In terms of nutritional value, dried karonda fruit exhibits higher energy content and elevated levels of specific antioxidants such as anthocyanins. It also boasts a rich mineral profile, including iron, calcium, zinc, magnesium, and potassium, all of which are beneficial for human health. These findings suggest that karonda fruit, an underutilized crop in Azad Jammu and Kashmir, Pakistan, can be effectively utilized in jam production, with the added advantage of being stored for up to 3 months without preservatives or deterioration. Overall, this study highlights the nutritional potential of karonda fruit and its suitability for producing flavorful and visually appealing jams enriched with valuable phytochemical compounds. Further, our results will provide guidance for the development of initial breeding material and program aimed at improving the nutritional value of karonda, which is one of the precious indigenous bioresources. The objective is to enhance its nutritional content and utilize it in the manufacturing of food products, addressing malnutrition and ensuring food security in the region.

## AUTHOR CONTRIBUTIONS

**Nagina Rafique:** Conceptualization (equal). **Turfa Mamoona:** Data curation (equal); formal analysis (equal). **Nazish Ashraf:** Writing – review and editing (equal). **Sheraz Hussain:** Investigation (equal). **Faisal Ahmed:** Methodology (equal). **Tawaf Ali Shah:** Writing – original draft (equal). **Amare Bitew Mekonnen:** Visualization; validation; software; writing – review and editing (equal). **Mohammed Bourhia**: reviewing and editing, investigations. **Ahmad Mohammad Salamatullah**: Investigations, data validation.

## CONFLICT OF INTEREST STATEMENT

There is no conflict of interest among authors.

## Data Availability

Data will be available from the corresponding author upon reasonable request.
